# Effect of Isothermal Repetitive Upsetting Extrusion on the Microstructure of Mg-12.0Gd-4.5Y-2.0Zn-0.4Zr Alloy

**DOI:** 10.3390/ma11112092

**Published:** 2018-10-25

**Authors:** Guanshi Zhang, Zhimin Zhang, Yue Du, Zhaoming Yan, Xin Che

**Affiliations:** School of Materials Science and Engineering, North University of China, Taiyuan 030051, China; s1403035@st.nuc.edu.cn (G.Z.); zhanggs2014@163.com (Y.D.); s1503017@st.nuc.edu.cn (Z.Y.); s1603090@st.nuc.edu.cn (X.C.)

**Keywords:** Mg-Gd-Y-Zn-Zr alloy, RUE, microstructure, DRX

## Abstract

Repetitive upsetting extrusion (RUE) was applied to the as-homogenized Mg-12.0Gd-4.5Y-2Zn-0.4Zr (wt %) alloy at 773 K. The microstructure evolution of the alloy during RUE was investigated. The results indicated that almost all Mg_5_(Gd,Y,Zn) phases and fine-lamellar long-period stacking-ordered (LPSO) phases were dissolved into the matrix after homogenization treatment at 793 K for 16 h. After one RUE pass, dynamic recrystallization (DRX) occurred. During subsequent RUE passes (from one to three passes), average volume fractions of DRXed grains increased from 43.9% to 65.8%, and that of fine-lamellar and block-shaped LPSO phases gradually decreased. All samples exhibited a typical bimodal microstructure consisting of some initial grains containing fine-lamellar LPSO phases, but consisting mostly of fine-DRXed grains with a mean grain size of 6 μm. Because of an increase in the accumulated strains, the coarse grains were substituted with fine-DRXed grains, the grains were gradually refined, and the microstructure distribution became more homogeneous.

## 1. Introduction

Mg and its alloys have attracted extensive attention in recent years as the most promising lightweight component materials. This is due to their superior mechanical properties, low density, good creep resistance, and excellent damping capacity. They have enormous potential in the aerospace, transportation, and 3C industries [1,2,3,4,5]. Diverse novel long-period stacking-ordered (LPSO) phases form in Mg-Gd-Y-Zr alloys with an additional element of Zn [6]. Compared with the magnesium matrix, LPSO phases exhibit higher thermal stability [7], elastic modulus [8], and microhardness [9]. A Mg_97_Zn_1_Y_2_ (at %) alloy with excellent strength of above 600 MPa at ambient temperature was successfully produced by rapidly solidified powder metallurgy processing (RS P/M) [10]. Its superior mechanical properties were attributed to ultrafine hcp-Mg grains ranging in size from 100 to 200 nm and to the fine-lamellar LPSO phase. This indicates that improving microstructures can result in an alloy with excellent performance.

Usually, increasing strains during severe plastic deformation (SPD) can lead to the original grains being completely substituted with grains that have undergone dynamic recrystallization (DRX) [11]. When the strains increase, more fine grains nucleate at the boundaries of the newly-formed fine grains. In this way, recrystallized fine grains form and broaden as the DRX process continues. Eventually, the recrystallized fine grains substitute with the original grains. Up to the present, several SPD processing techniques have been developed on magnesium alloys (e.g., high-pressure torsion (HPT) [12], cyclic extrusion and compression (CEC) [13], friction stir processing (FSP) [14], equal-channel angular pressing (ECAP) [15], and severe rolling (SR) [5]). Aizawa and Tokumitu found that repetitive upsetting extrusion (RUE) was a promising SPD technique for grain refinement and property improvement in bulk materials [16]. The RUE process has several advantages: (1) each pass is composed of the upsetting and extrusion processes, which allows multipass deformation in order to accumulate high plastic strains; (2) grain size is significantly refined; (3) elongation of the alloys noticeably increases; (4) microstructure distribution becomes more homogeneous; and (5) yield strength of the alloys increases. Hu et al. investigated grain refinement mechanisms for LY12 aluminum alloy during RUE [17], concluding that it is easy to achieve high accumulative induced strain using the desired RUE cycles. Following this, the fine uniform structure was obtained. Xu et al. studied the microstructure and mechanical properties of AZ61 alloy processed by RUE [18], finding that after three RUE passes at 558 K the mean grain size was dramatically refined to 3.5 μm and that mechanical properties markedly improved.

In the present study, RUE was applied to achieve SPD, and the evolution of isothermal RUE on the microstructure of Mg-12.0Gd-4.5Y-2.0Zn-0.4Zr alloy was investigated.

## 2. Materials and Methods

A high-quality Mg-12.0Gd-4.5Y-2Zn-0.4Zr (wt %) alloy 3000 mm in length and 330 mm in diameter was prepared by Wenxi YinGuang of Magnesium Industry (Group) Co., Ltd., (Wenxi, China). Cylindrical samples 25 mm in height and 10 mm in diameter were cut from the middle of the as-cast rod. These samples were homogenized at 793 K for 16 h in a high-temperature heat treatment furnace (Nabertherm N15/65HA) and then quenched in water at about 343 K.

**Figure 1:**
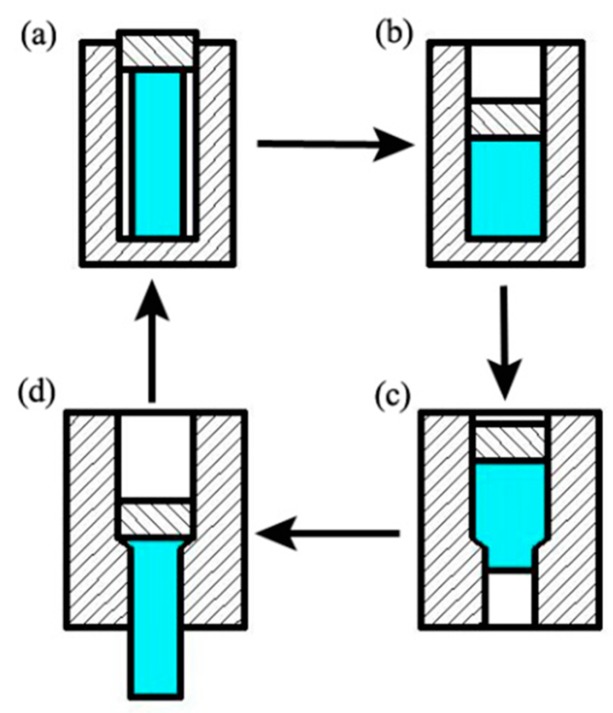
Schematic diagrams of repetitive upsetting extrusion (RUE): (**a**) the upsetting process; (**b**) the end of the upsetting process; (**c**) the extrusion process; and (**d**) the end of the extrusion process.

The diameters of the upsetting and extrusion cavities were 14 mm and 10 mm, respectively. The accumulated strain was ε = 2 ln(d_1_/d_2_) = 0.6729 (where d_1_ is the diameter of the upsetting cavity and d_2_ is the diameter of the extrusion cavity) during each upsetting or extrusion process. To achieve thermal stability, prior to RUE the as-homogenized cylindrical samples and the dies were held in the resistance furnace at 773 K for 10 min and 60 min, respectively. Afterwards, they were processed isothermally at 773 K using an Instron 3382 testing machine with a ram speed of 2 mm/min. Oil-based graphite was attached to the surface of each sample to minimize the friction. The as-homogenized samples were first subjected to the upsetting process and then the extrusion process. The RUE samples and dies were reheated at 773 K for 10 min after each RUE pass. After the final pass, the samples were quenched in cold water. The RUE processing schematic used in the present study is shown in Figure 1.

Before microstructure observation, the RUE samples were machined perpendicularly to the extrusion direction. The samples were mechanically polished and chemically etched in a solution of 6 mL distilled water, 2 g picric acid, 40 mL ethanol, and 4 mL acetic acid. The phases in the alloy were identified by X-ray diffraction (XRD) on a diffractometer (DX-2700, Dandong, China). XRD data was detected in the 2 θ diffraction angle ranges from 25 to 85 degrees at a scanning speed of 5 deg/min. The microstructure was investigated using optical microscopy (OM, Zeiss, Oberkochen, Germany) and scanning electron microscopy (SEM, Hitachi SU5000, Tokyo, Japan), with an accelerated voltage of 20 kV both prior to and after RUE. The chemical compositions of the phases were analyzed using an energy-dispersive X-ray spectrometer (EDS, EDAX Inc., Mahwah, NJ, USA). Electron backscattered diffraction (EBSD) orientation maps were obtained at 20 kV, a tilt angle of 70°, a 15 mm working distance, and a scan step of 0.2 μm by an EDAX-TSL (EDAX Inc., Mahwah, NJ, USA) EBSD. The EBSD data were analyzed by TSL OIM analysis software (version 7.3, EDAX Inc., Mahwah, NJ, USA).

## 3. Results and Discussion

### 3.1. The Microstructures of the As-Cast and As-Homogenized Alloys

**Figure 2:**
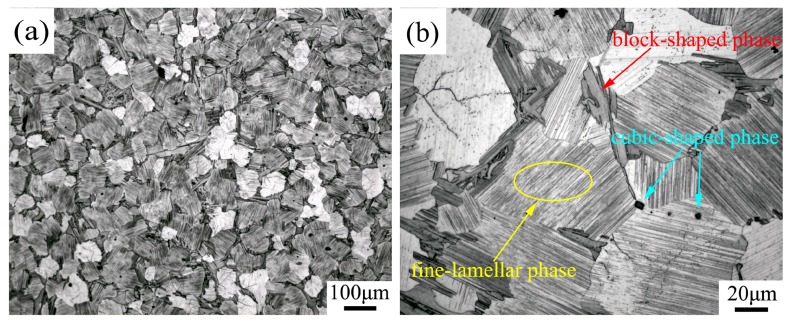
Optical microscopy (OM) images of: (**a**) the as-cast alloy; and (**b**) the corresponding magnified image.

Figure 2a depicts the OM image of an as-cast alloy. The interdendritic phases are mostly distributed along grain boundaries, with a mean grain size of about 70 μm. Three different phases can be observed in the OM image in Figure 2b: A fine-lamellar phase, a block-shaped phase, and a cubic-shaped phase. The fine-lamellar phases seen in the OM image were distributed from grain boundaries to the interior of the large grains and had the same orientation relationship in one grain. The block-shaped phases were distributed along the grain boundaries. The cubic-shaped phases were distributed discontinuously at grain boundaries and in the interior of the grains.

**Figure 3:**
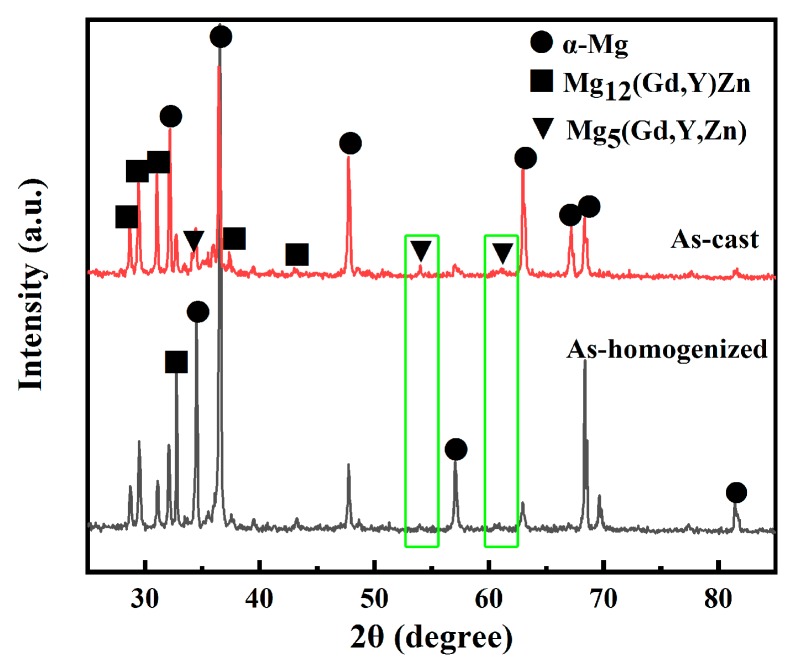
XRD patterns of Mg-12.0Gd-4.5Y-2Zn-0.4Zr in as-cast and as-homogenized states.

XRD and backscattered electron (BSE)-SEM observations were used to further investigate the microstructure of the as-cast alloy. The XRD pattern of the as-cast alloy is presented in Figure 3. Three phases were detected: α-Mg, Mg_12_(Gd,Y)Zn, and Mg_5_(Gd,Y,Zn). Previously, others already proved that the Mg_12_(Gd,Y)Zn phase is an LPSO phase [19].

**Figure 4:**
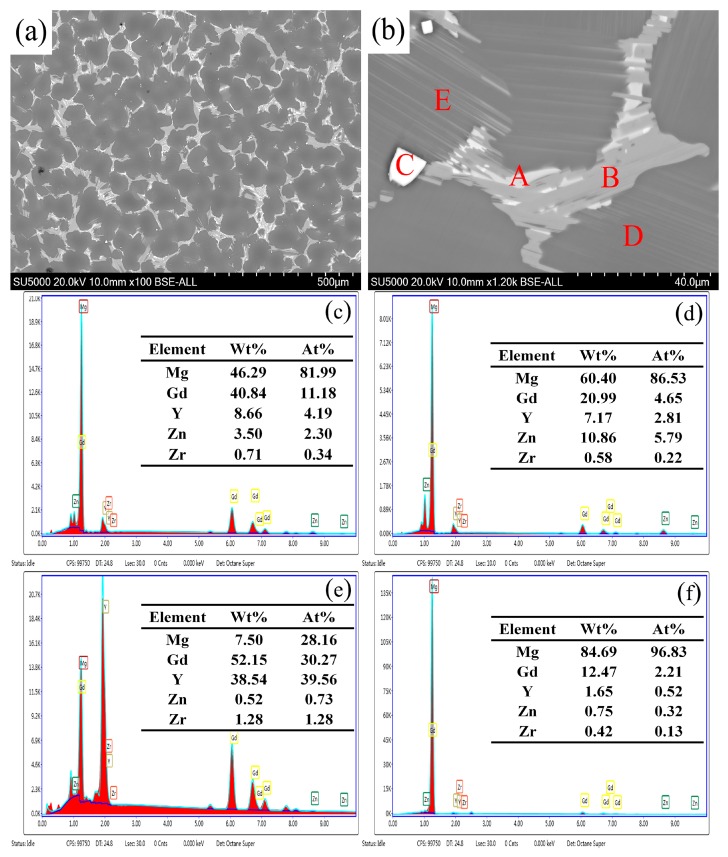
The microstructure of the as-cast Mg-12.0Gd-4.5Y-2Zn-0.4Zr alloy: (**a**) backscattered electron-scanning electron microscopy (BSE-SEM); (**b**) a magnified image of (**a**); and (**c**–**f**) the corresponding energy-dispersive X-ray spectrometer (EDS) results of the phases marked A, B, C, and D, respectively.

The BSE-SEM micrographs of the as-cast alloy and corresponding EDS results of the phases are shown in Figure 4. The chemical composition of the grayish-shaped phase (Figure 4b, point A) was Mg-11.18Gd-4.19Y-2.30Zn-0.34Zr (at %) (Figure 4c), which indicates that the stoichiometry of this phase was near Mg_5_(Gd,Y,Zn). The chemical composition of the block-shaped phase (Figure 4b, point B) was Mg-4.65Gd-2.81Y-5.79Zn-0.22Zr (at %) (Figure 4d), which indicates that this phase’s stoichiometry was near Mg_12_(Gd,Y)Zn, the same as an LPSO structure. The cubic-shaped phase (Figure 4b, point C) could be considered to be an RE-rich compound due to the EDS results (Figure 4e). However, the RE-rich compound could not be analyzed by XRD due to its low-volume fractions. Furthermore, the fine-lamellar phase (Figure 4b, point E) emerged inside α-Mg (Figure 4b, point D) grains, which should be an LPSO phase.

**Figure 5:**
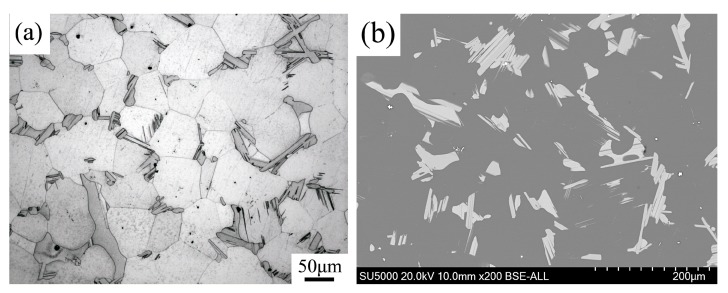
The microstructure of the Mg-12.0Gd-4.5Y-2Zn-0.4Zr alloy after homogenization: (**a**) OM; and (**b**) BSE-SEM micrographs.

Figure 5 depicts OM and BSE-SEM microstructures of samples homogenized at 793 K for 16 h. The volume fractions of the interdendritic phases along the grain boundaries decreased. Almost all fine-lamellar phases were dissolved into the α-Mg matrix. As seen in Figure 3, after homogenization treatment some diffraction peaks in the as-cast alloy Mg_5_(Gd,Y,Zn) disappeared (marked by the green frame). It can be concluded that almost all Mg_5_(Gd,Y,Zn) phases were dissolved into the matrix. Only some block-shaped LPSO phases remained along the grain boundaries, primarily at triple junctions (Figure 5b).

### 3.2. Load-Displacement Curves

**Figure 6:**
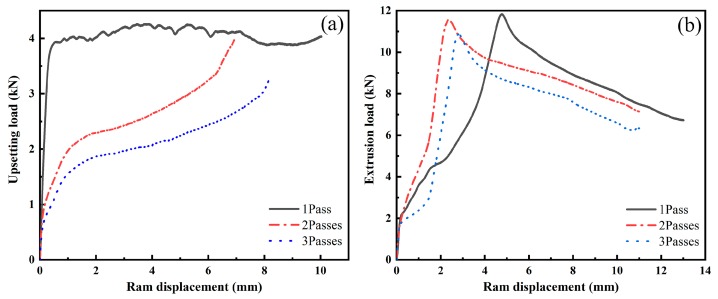
Load-displacement curves for individual: (**a**) upsetting; and (**b**) extrusion processes corresponding to one, two, and three passes, respectively.

Figure 6 depicts representative load-displacement curves for the individual upsetting and extrusion processes corresponding to one, two, and three passes, respectively. At the beginning stage of the upsetting process (Figure 6a), the load rapidly increased and gradually reached a steady state. Following increased ram displacement, the contact area between the external surface of the sample and the die inner wall grew. The upsetting load gradually increased because of limited radial flow of the metal. The curves indicate that DRX occurred in the Mg-12.0Gd-4.5Y-2.0Zn-0.4Zr alloy during each upsetting procedure, which resulted in a dynamic balance between the work-hardening and DRX-softening processes.

At the beginning stage of the extrusion process (Figure 6b), the load curve first shows a steady increase, reaching a maximum, but dropping with increased ram displacement. Figure 6 shows that the load of the extrusion processes was significantly greater than that of the upsetting deformations because the sample was subjected to high hydrostatic compressive stress during the extrusion process.

Figure 6a shows that with increasing RUE passes, the load in the steady state was obviously reduced during the upsetting process, displaying a downward displacement of the load-displacement curve. At the same time, the maximum load reduction efficiency gradually decreased. When the RUE passes increased to three passes, the steady state load reached a minimum of 2 kN. The load-displacement curves of the extrusion process displayed in Figure 6b follow a similar trend in that the curve slopes downward because the deformation load gradually decreased with increasing RUE passes. The maximum load of extrusion fell to a minimum of 11 kN after three RUE passes. Because of an increase in the accumulated strains, the grains of the alloy were refined gradually, which led to greater plasticity and lower deformation reinforcement at high temperature.

### 3.3. Microstructure Evolution

**Figure 7:**
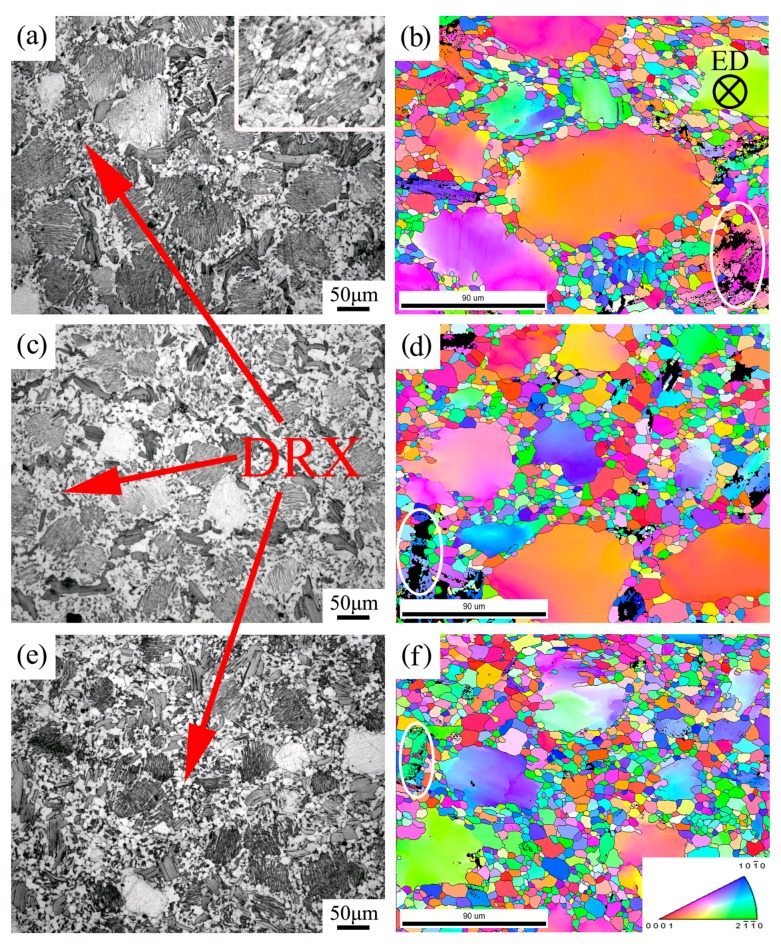
Microstructure observations obtained from the transverse cross-sections of the RUE-formed Mg-12.0Gd-4.5Y-2Zn-0.4Zr alloy: (**a**,**b**) one pass; (**c**,**d**) two passes; (**e**,**f**) three passes; (**a**,**c**,**e**) OM images; and (**b**,**d**,**f**) electron backscattered diffraction (EBSD) orientation maps. The color key triangle shows the crystallographic orientation of hcp crystal.

The OM of the transverse cross-sections of the samples are presented in Figure 7a,c,e, which corresponds to one, two, and three RUE passes, respectively; Figure 7a,c,e thus displays the changes that occurred during the distribution and morphology of the phases. The microstructure evolution during different RUE passes can be seen in EBSD orientation maps obtained from the transverse cross-sections, as seen in Figure 7b,d,f. The large black regions in the maps, marked by white circles, correspond to LPSO phases because of the Kikuchi diffraction patterns were not recognized by the orientation image mapping (OIM) system [20]. As seen in Figure 7a,b, after one RUE pass, the accumulated strains reached 1.35, and many coarse initial grains remained. A few fine grains appeared in the interior of the deformed grains, as shown in the magnified OM image (Figure 7a), indicating DRX occurred. Numerous fine-DRXed grains were distributed along the grain boundaries. In addition, the contrast of some deformed grains was dark, revealing that dense fine-lamellar LPSO phases emerged in the interior of the grains. Meanwhile, several fine-lamellar phases were bent or kinked to some degree. Many dislocations formed inside the grains, some of them decomposing into stacking faults (SF) during RUE. At the same time, the solid-solution atoms in the matrix rearranged to meet the chemical conditions and structural conditions of the LPSO structure, which contributed to the appearance of the LPSO phases in the interior of the grains.

With increasing RUE passes (from one to three), the average volume fraction of DRXed grains gradually increased from 43.9% to 65.8%. All the samples exhibited a typical bimodal microstructure consisting of some initial grains containing fine-lamellar LPSO phases, but consisting mostly of fine-DRXed grains with a mean grain size of 6 μm. It should be noted that the mean size of the fine grains did not significantly change. It was also found that the kink of the LPSO phases could still be observed and that it induced the occurrence of DRX.

RUE combines the traditional upsetting and extrusion processes to achieve high accumulated strains. The main grain refinement mechanism is the DRX process. Obvious color variation can be observed in some coarse grains, as seen in Figure 7b,d,f, which indicates the occurrence of misorientation induced by subgrain boundaries and dense dislocations in these deformed grains. Most dislocations caused by RUE provide the driving force for dynamic recovery and DRX. Here, because of the increase in the accumulated strains, the dislocation density increased and the dislocations tangled with each other. When the accumulated strains reached a critical level, the dislocations rearranged, propagated, and formed subgrain boundaries. Because of the increase in the accumulated strains, dislocation reactions became more critical. Subgrain boundaries then converted to high-angle boundaries, which led to the formation of new grains.

At the beginning stage of RUE, the fine-lamellar LPSO phases in the interior of the grains and the block-shaped LPSO phases along the grain boundaries had an inhibition effect on the movement of dislocations and the growth of DRXed grains. A small number of DRXed grains occurred along the grain boundaries and split the initial grain into several parts, which became the main grain refinement characteristic of this stage. However, the accumulated strain was only 0.67, which cannot provide a force sufficient to complete the DRX process. Consequently, only incomplete DRX occurred. This then led to the occurrence of incomplete static recrystallization because of heating and holding during the interpass. At the same time, the fine-lamellar LPSO phases effectively prevented the occurrence of static recrystallization and grain growth, which led to the retention of the fine-DRXed grain area that had existed in previous passes.

Because of an increase in the accumulated strains, the coarse grains were substituted with fine-DRXed grains, the grains were gradually refined, and the microstructure distribution became more homogeneous. Due to the accommodation-deformation effect of the fine-DRXed region, the deformation degree of coarse grain and the number of kink bands of fine-lamellar LPSO phases decreased. Furthermore, the block-shaped LPSO phases were also broken, which reduced the inhibition effect on the movement of dislocations. In addition, the deformed grains exhibited an almost ellipsoidal shape. This was primarily due to RUE causing the grains to be stretched in both the radial and axial directions.

The thermal deformation process caused the appearance of fine-lamellar LPSO phases in the interior of grains; this can coordinate the deformation. LPSO phases can also remain in the deformed grain at high temperatures during different RUE passes. It can be concluded that LPSO phases have a higher thermal stability. Because of an increase in the accumulated strains, the DRXed grain areas were gradually expanded, accompanied by a decrease of volume fractions in the fine-lamellar and block-shaped LPSO phases.

## 4. Conclusions

- The microstructure of an as-cast alloy consists of α-Mg grains, grayish Mg_5_(Gd,Y,Zn) phases, block-shaped LPSO phases, a cubic-shaped RE-rich compound, and fine-lamellar LPSO phases. After homogenization treatment at 793 K for 16 h, almost all Mg_5_(Gd,Y,Zn) phases and fine-lamellar LPSO phases dissolved into the matrix. Only some block-shaped LPSO phases remained along the grain boundaries, primarily at triple junctions.
- At the beginning stage of the upsetting process, the load rapidly increased and gradually reached a steady state. When the RUE passes increased to three passes, the steady state load reached a minimum of 2 kN. At the beginning stage of the extrusion process, the load curve first shows a steady increase, reaching a maximum, but then dropping with increasing ram displacement. The maximum load of extrusion fell to a minimum of 11 kN after three RUE passes.
- After one RUE pass, a few DRXed grains appeared in the interior of the deformed grains and along grain boundaries. From one to three RUE passes, average volume fractions of DRXed grains increased from 43.9% to 65.8%, and that of fine-lamellar and block-shaped LPSO phases gradually decreased.
- All the samples exhibited a typical bimodal microstructure consisting of some initial grains containing fine-lamellar LPSO phases, but consisting mostly of fine-DRXed grains with a mean grain size of 6 μm. Because of an increase in the accumulated strains, the coarse grains were substituted with fine-DRXed grains, the grains were gradually refined, and the microstructure distribution became more homogeneous.
